# A novel risk model based on cuproptosis-related lncRNAs predicted prognosis and indicated immune microenvironment landscape of patients with cutaneous melanoma

**DOI:** 10.3389/fgene.2022.959456

**Published:** 2022-07-22

**Authors:** Yi Zhou, Qi Shu, Zailin Fu, Chen Wang, Jianrong Gu, Jianbo Li, Yifang Chen, Minghua Xie

**Affiliations:** ^1^ Department of Pharmacy, First People’s Hospital of Linping District, Hangzhou, ZG, China; ^2^ The Cancer Hospital of the University of Chinese Academy of Sciences (Zhejiang Cancer Hospital), Institute of Basic Medicine and Cancer (IBMC), Chinese Academy of Sciences, Hangzhou, China

**Keywords:** cutaneous melanoma, cuproptosis-related lncRNA, risk model, immune microenvironment, prognosis

## Abstract

Cutaneous melanoma (CM) is an aggressive form of malignancy with poor prognostic value. Cuproptosis is a novel type of cell death regulatory mechanism in tumors. However, the role of cuproptosis-related long noncoding RNAs (lncRNAs) in CM remains elusive. The cuproptosis-related lncRNAs were identified using the Pearson correlation algorithm. Through the univariate and multivariate Cox regression analysis, the prognosis of seven lncRNAs associated with cuproptosis was established and a new risk model was constructed. ESTIMATE, CIBERSORT, and single sample gene set enrichment analyses (ssGSEA) were applied to evaluate the immune microenvironment landscape. The Kaplan–Meier survival analysis revealed that the overall survival (OS) of CM patients in the high-risk group was remarkably lower than that of the low-risk group. The result of the validated cohort and the training cohort indicated that the risk model could produce an accurate prediction of the prognosis of CM. The nomogram result demonstrated that the risk score based on the seven prognostic cuproptosis-related lncRNAs was an independent prognostic indicator feature that distinguished it from other clinical features. The result of the immune microenvironment landscape indicated that the low-risk group showed better immunity than high-risk group. The immunophenoscore (IPS) and immune checkpoints results conveyed a better benefit potential for immunotherapy clinical application in the low-risk groups. The enrichment analysis and the gene set variation analysis (GSVA) were adopted to reveal the role of cuproptosis-related lncRNAs mediated by the immune-related signaling pathways in the development of CM. Altogether, the construction of the risk model based on cuproptosis-related lncRNAs can accurately predict the prognosis of CM and indicate the immune microenvironment of CM, providing a new perspective for the future clinical treatment of CM.

## Introduction

Cutaneous melanoma (CM) is recognized as one of the deadliest forms of skin cancer (Arnold et al., 2022). Due to its high rate of invasion and distant metastasis, CM accounts for 72% of mortality among skin cancer patients (Rebecca et al., 2020). Recently, immune checkpoint blockade (ICB) has displayed remarkable clinical benefits in various cancers, including lymphoma, melanoma, and squamous non-small cell lung cancer (NSCLC) (Alencar and Moskowitz, 2019). Although ICB is effective in a variety of cancer types, and most are not restricted by certain gene mutation statuses, only a few patients have successfully produced a complete response. Therefore, the complete revelation of the mechanism related to this phenomenon is vital.

Copper is an essential cofactor in every organism and copper chelators have been suggested as anticancer agents (Zheng et al., 2022). However, the exploitation of copper toxicity on tumors has shown an unsatisfactory result. A recent study by Brady et al. pointed that cell death can be a consequence of copper exceeding the homeostasis threshold (Brady, 2022). Unlike all other known mechanisms of cell death regulation, including apoptosis, ferroptosis, pyroptosis, or necrosis, this newly-discovered copper-dependent regulatory cell death (so-called cuproptosis) relies on mitochondrial respiration (Tsvetkov et al., 2022). Copper binds directly to the lipid components of the tricarboxylic acid (TCA) cycle, leading to lipoylated protein aggregation and a subsequent loss of iron-sulfur cluster proteins, resulting in proteotoxic stress and ultimately cell death (Tardito et al., 2011). Elesclomol, a copper ion carrier, has shown its impact as a new form of oxidative stress inducer on tumor cells which triggers their apoptosis (Oliveri, 2022). Although a phase 3 combination clinical trial of elesclomol in patients with melanoma showed a lack of efficacy in the unselected population, its anti-tumor effect was demonstrated in a post hoc analysis of the patients with low plasma levels of lactate dehydrogenase (LDH), reflecting a trend towards a higher mitochondrial metabolism dependency (Asdourian et al., 2022). This finding prompts the role of cuproptosis in melanoma patients with low LDH levels and could be further exploited by taking advantage of personalized precision medicine. However, current research on the effects and mechanisms of cuproptosis is still in its infancy.

Long non-coding RNAs (lncRNAs) are defined as transcripts with over 200 nucleotides in length without evident protein-coding function (Leucci et al., 2016). It is known to play a vital role in many aspects of gene expression during physiological processes and disease progression (Hulstaert et al., 2017). The presence of abnormal expressed lncRNAs is detected in various diseases, including metabolic diseases, cardiovascular diseases, and tumors (De Falco et al., 2021). Various lncRNAs have been confirmed to be involved in multiple diseases and certain lncRNAs have been identified as disease biomarker (Ma et al., 2017). In CM, several lncRNAs are strongly associated with the development of malignant characteristics, including cell cycle arrest, inhibitory tumor microenvironment formation, tumoral cellular signal pathway activation, and poor prognosis relevance (Xue et al., 2021; Zeng et al., 2022). However, the role of cuproptosis-associated lncRNAs in CM remains elusive.

Numerous studies have provided evidence that tumor progression and recurrence are shaped by the tumor microenvironment (TME) in addition to the inherent genetic changes of cancer cells (Ruocco et al., 2022). The interaction between tumor cells and the microenvironment leads to a tumor-driven immune response. Previous studies have shown that complex TME, particularly the tumor immune microenvironment (TIME), is the main factor contributing to the poor prognosis of CM patients (Romano et al., 2021). During melanoma genesis, both tumor cell proliferation and apoptosis are influenced by the activity of immune cells (Zhou et al., 2021). Malignant cells evade host immunity and change the TME milieu of CM patients through the activation of immune checkpoints. Although ICB therapy targeting the *PD-1/PD-L1* axis and cytotoxic T lymphocyte-associated antigen-4 (*CTLA-4*) has shown dramatic improvement in survival rate for many CM patients (Khunger et al., 2019), meaningful clinical responses have only been observed in a relatively small subset of patients. With only these factors in mind, it is impossible to accurately predict the successful outcome of CM patients who receives ICB treatment. However, recent studies revealed the significance of lncRNAs in TIME. In addition to directly regulating immune cells, lncRNAs could also regulate inflammation and participate in immune gene expression, hence influencing TIME (Ma et al., 2022). Nevertheless, the link between cuproptosis-related lncRNAs and TIME in CM is still widely unknown.

In this study, following the analysis of The Cancer Genome Atlas (TCGA) database, we systematically investigated the association between cuproptosis-related lncRNAs and the clinicopathological features of CM patients. A novel risk model was constructed based on seven cuproptosis-related lncRNAs, and the ability of cuproptosis-related lncRNAs to predict the prognosis of patients with CM was assessed. Moreover, the immune microenvironment of CM patients was comprehensively analyzed, and the possible signaling pathways involved were explored in detail. In conclusion, the results of this study provide new perspectives and insights regarding the potential strategies for managing and treating CM patients.

## Materials and methods

### Data collection

**FIGURE 1:**
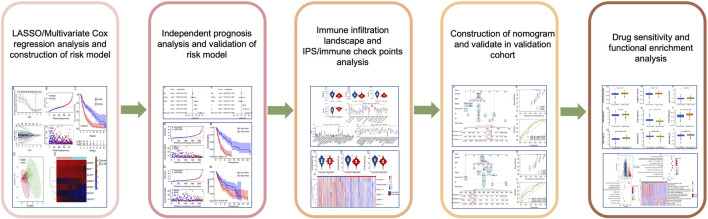
Study flow diagram.

The transcriptome matrix and clinical characteristic materials were obtained from The Cancer Genome Atlas database (TCGA) (https://portal.gdc.cancer.gov/) (Wu J. et al., 2022). In this study, the criterion for sample inclusion was survival time great than 0, and a total of 454 samples were included in the present study. Then, the gene expression matrix of each CM patient was merged using Perl scripts. The expression of mRNA and lncRNAs were annotated and classified using the ensembles human genome browser GRCh38.p13 (http://asia.ensembl.org/index.html). The clinical characteristic materials included survival time, survival status, age, gender, stage, and T, N stage were extracted using Perl scripts from the TCGA database. Due to the samples size of CM patients in M stage was greatly different, so it was excluded in the analysis. All data and clinical information involved in this study were obtained from the public database. Approval from the ethics committee and written informed consent from patients were not required. The process of data analysis was shown in Figure 1.

### Identification of cuproptosis-related long noncoding RNAs

The cuproptosis genes in this study were collected from a previous study (Tsvetkov et al., 2022). Perl scripts were applied to extract the cuproptosis gene expression of CM, and the Pearson correlation analysis was performed to identify the cuproptosis-related lncRNAs. With the threshold setting at |correlation coefficient| > 0.4, *p*-value < 0.001 (r > 0.4, *p* < 0.001) (Lin et al., 2020), and a total of 495 cuproptosis-related lncRNAs were identified (Supplementary Table S1).

### Construction of risk model

According to the univariate Cox regression analysis, the least absolute shrinkage and selection operator (LASSO) algorithm was conducted to identify the cuproptosis-related lncRNAs associated with overall survival rate using R package “glmnet”. Then, multivariate Cox regression analysis was used to identify the candidate prognostic cuproptosis-related lncRNAs and constructed the risk model. The risk scores of each sample were calculated according the following formula: = (-0.19 x LINC01150 expression) + (-0.06 x EBLN3P expression) + (-0.02 x MIR100HG expression) + (0.07 x AC009495.1 expression) + (-0.04 x WAC-AS1 expression) + (-0.03 x LINC00339 expression) + (-0.18 x USP30-AS1 expression). According to the median risk score, the samples were divided into low- and high-risk groups. The Kaplan-Meier survival curve was conducted to estimate the difference of overall survival in low- and high-risk groups via log-rank algorithm. The principal component analysis (PCA) was performed to investigate the separation pattern based on the prognonstic cuproptosis-related lncRNAs between the low- and high-risk groups using R package “ggplot2”. According to the prognostic cuproptosis-related lncRNAs, the samples were classified into the training cohort and the validation cohort to the ratio of 7:3, and calculated the risk score of each sample, respectively.

### Evaluation of risk model independence

Univariate and multivariate Cox regression analysis were performed to investigate the risk model was an independent indicator of patients with CM. A nomogram model was constructed using clinicopathological characteristic and risk scores with “rms” R package. According to Cox regression analysis, all variates were calculated and estimated the 1-, three- and five- year survival probability of patients, lower scores mean better prognosis. Calibration plots and consistency (C-index) was a commonly parameters to assess the accuracy of nomograms and the C-index was positively correlated with the nomogram accuracy. The prognostic capability of the risk model constructed by risk scores was validated using time-dependent receiver operating characteristic (ROC) analysis. The calibration plot was another parameter to verify the accuracy of the nomogram, a straight line closer to 45 degrees means better predictive power.

### Immune microenvironment landscape analysis

ESTIMATE algorithm was conducted to evaluate the estimation of stromal and immune cells in tumor tissues. Stromal, immune, ESTIMATE scores and tumor purity were calculated via “estimate” R package. CIBERSORT algorithm was used to investigate the immune infiltration landscape, and 22-types immune cells were evaluated using “CIBERSORT R script v1.03”. In addition, the immune function score of each sample was calculated by “estimate” R packages. A single sample gene set enrichment analysis (ssGSEA) algorithm was performed to assess the infiltration level of 23-types of immune cells via the “GSVA” R packages.

### Immunophenoscore and drug sensitivity analysis

Immunophenoscore (IPS) of each CM patient was obtained from the TCIA database (https://tcia.at/home). The expression of immune checkpoints included *BTLA, PDCD1LG2, PD-L1, CTLA4, LAG3*, and *PD-1* were extracted from the TCGA using Perl scripts. IC50 was an important indicator for evaluating drug efficacy or sample response to treatment. Based on the cancer Drug Sensitivity Genomics (GDSC), the frug treatment response of each patient with CM was predicted via R package “pRRophetic”.

### Functional enrichment analysis

The “limma” R package was used to identify the differential expression genes (DEGs), and the *p*-value was adjusted using “FDR” method. Moreover, the threshold for screening DEGs was set at |Fold Change| ≥ 2 and FDR <0.05. Biology process (BP) and Kyoto Encyclopedia of Genes and Genomes (KEGG) analysis were performed to enrich the DEGs into pathways using the “clusterProfiler” R package (Yu et al., 2012). Additionally, the activity of KEGG term in each patient with CM was conducted via GSVA using the “GSVA” R package.

### Statistical analysis

All statistical analyses were performed using R software (version 4.1.0). Differential functions were analyzed using the Wilcoxon rank-sum test between the two groups, and statistical significance was set at *p* < 0.05.

## Results

### Risk model construction of cuproptosis-related long noncoding RNAs

**FIGURE 2:**
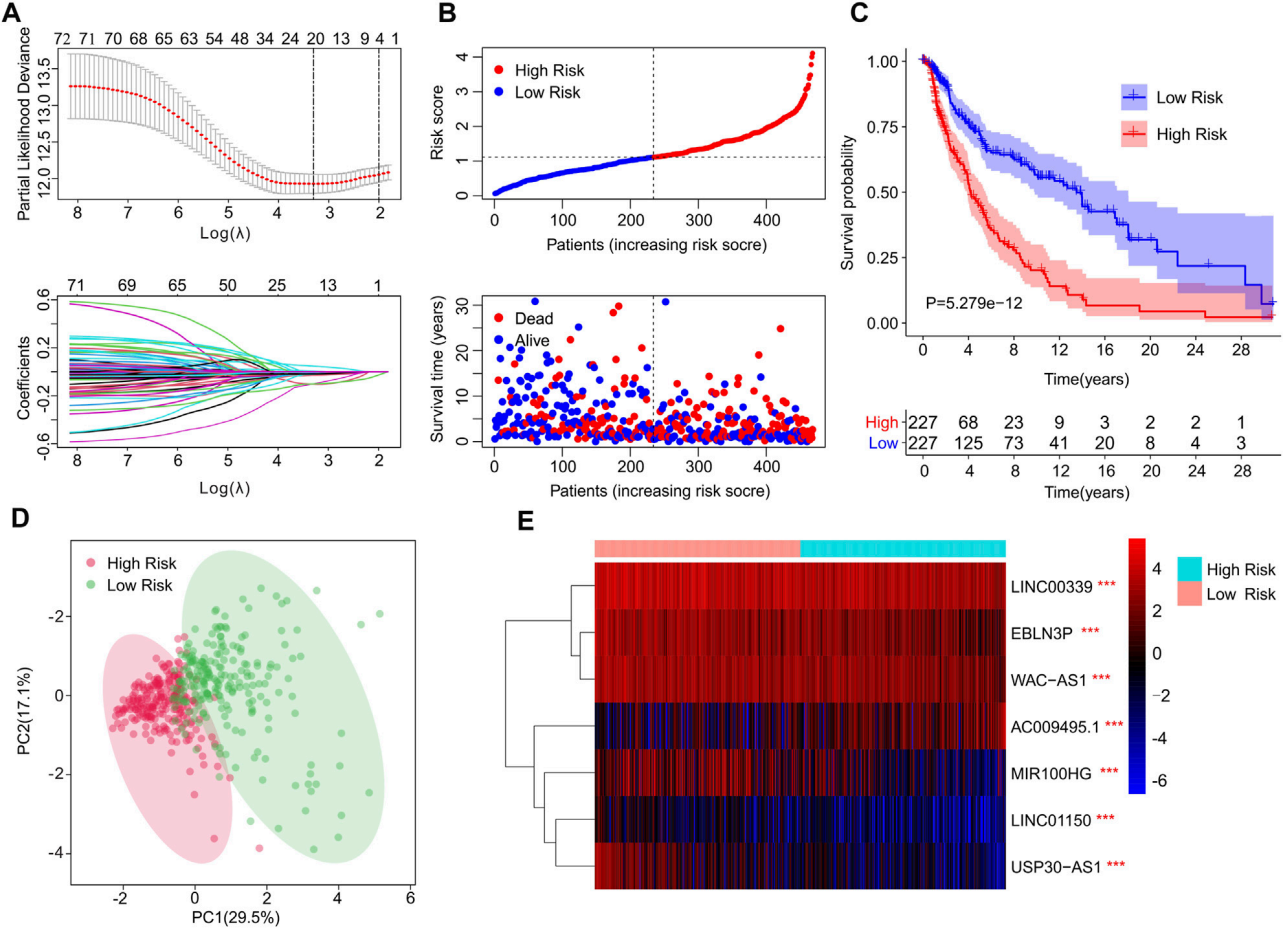
Risk model construction based on the prognostic cuproptosis-related lncRNAs of patients with CM. **(A)** LASSO showed the coefficients and minimal lambda of prognostic cuproptosis-related lncRNAs. **(B)** Distribution of risk scores of CM patients and scatter plot showed the correlation between cuproptosis-related lncRNAs prognostic features and survival time. **(C)** The Kaplan-Meier survival curve illustrated that the survival rate of patients in low-risk group was significantly longer than those in high-risk groups. **(D)** The principal component analysis (PCA) displayed a significant separation between low- and high-risk groups based on the seven prognostic cuproptosis-related lncRNAs. **(E)** The heatmap visual result demonstrated the expression of seven cuproptosis-related lncRNAs in low- and high-risk group.

A novel risk model was constructed to assess the prognosis of cuproptosis-related lncRNAs in CM. Based on the univariate Cox analysis, 20 cuproptosis-related lncRNAs associated with OS were identified via the least absolute shrinkage and selection operator (LASSO) analysis (Figure 2A). Among them, seven cuproptosis-related lncRNAs that, could independently predict the prognosis of patients with CM (Supplementary Figure S1), were selected to construct a risk model using multivariate Cox regression analysis. For the ensuing analysis, the CM patients were ranked using risk scores and classified into low- and high-risk groups by dichotomization. The scatter dot plot demonstrated the survival time of each patient was closely related to their risk score (Figure 2B). The Kaplan-Meier survival curve showed that patients in the low-risk group have a significant survival advantage in comparison with high-risk group patients (Figure 2C). Principal component analysis (PCA) displayed a clear distinction between low- and high-risk groups based on the seven cuproptosis-related lncRNAs (Figure 2D). The heatmap visualization result demonstrated differences in the expression of seven cuproptosis-related lncRNAs between low- and high-risk groups. The low-risk groups revealed higher expressions of LINC01150, EBLN3P, MIR100HG, WAC−AS1, LINC00339, and USP30−AS1, but a lower expression of AC009495.1 (Figure 2E).

### Risk model based on cuproptosis-related long noncoding RNAs was an independent prognosis factor

**FIGURE 3:**
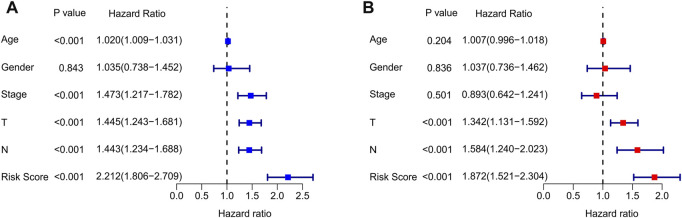
Independent prognosis analysis of clinical characteristics and risk score. **(A)** Univariate Cox regression analysis showed the association between the overall survival rate and clinical characteristics include age, gender, stage, T, N, and the cuproptosis-related lncRNAs prognostic signature risk scores. **(B)** Multivariate Cox regression analysis demonstrated that T, N, and risk score are independent prognostic indicator for overall survival rate of CM patients.

**FIGURE 4:**
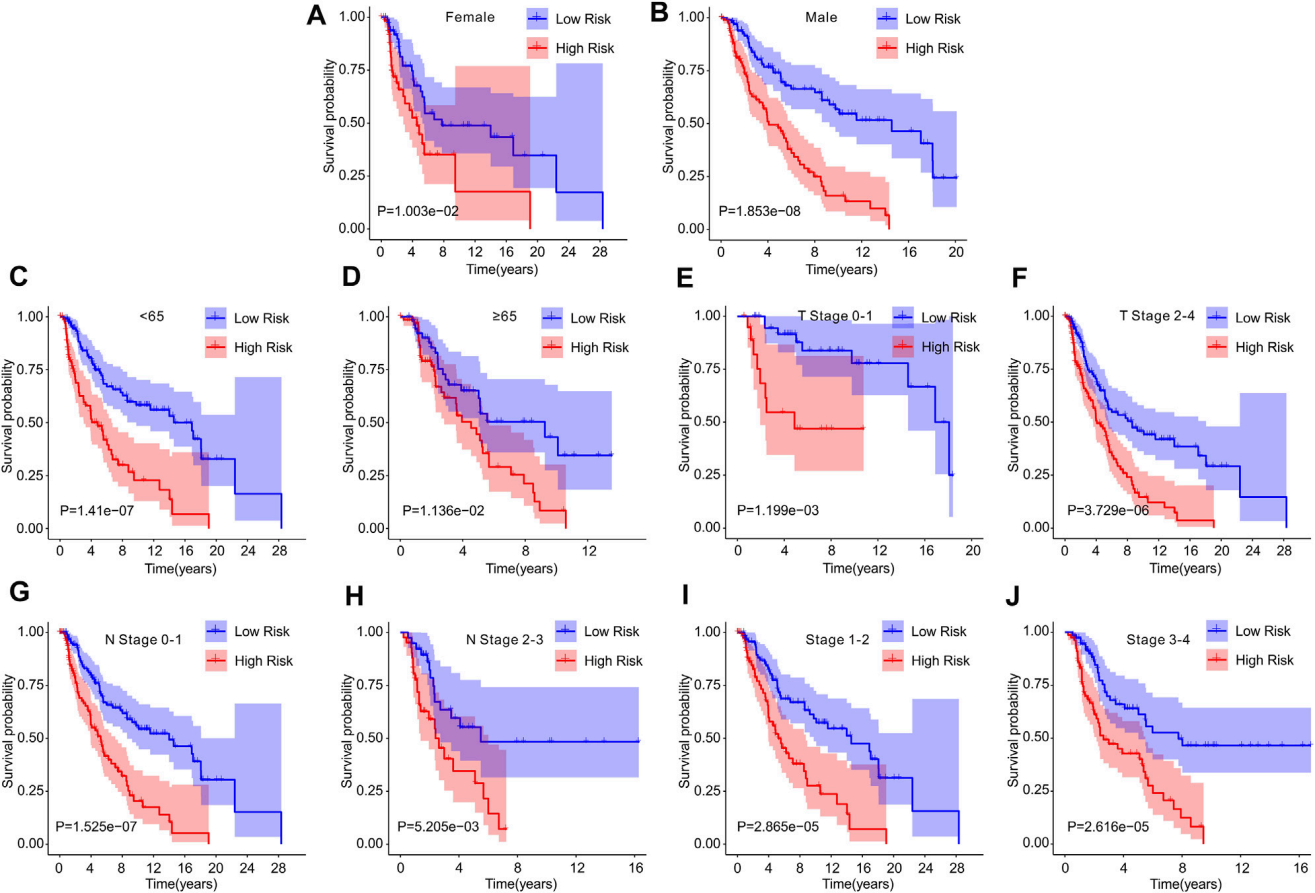
The Kaplan-Meier survival curve of patients in low- and high-risk groups stratified by different clinicopathological characteristics. The survival curve showed the overall survival rate of patients in low- and high-risk groups stratified by **(A,B)** gender (female vs male), **(C,D)** age (<65 vs ≥ 65), **(E,F)** T stage (T 0-1 vs T 2-4), **(G,H)** N stage (N 0-1 vs N 2-3), **(I,J)** stage (stage 1-2 vs stage 3-4).

Univariate Cox analysis and multivariate Cox analysis were further performed to investigate the cuproptosis-related lncRNAs as an independent prognostic indicator for CM. Univariate Cox analysis suggested that age (hazard ratio (HR) = 1.020, *p* < 0.001), stage (HR = 1.473, *p* < 0.001), T stage (HR = 1.445, *p* < 0.001), N stage (HR = 1.443, *p* < 0.001), and risk score (HR = 2.212, *p* < 0.001) were all closely related to the OS of CM patients (Figure 3A, Supplementary Table S1). Multivariate Cox analysis revealed that T stage (HR = 1.342, *p* < 0.001), N stage (HR = 1.584, *p* < 0.001), and risk score (HR = 1.872, *p* < 0.001) were substantially associated with OS (Figure 3B, Supplementary Table S2). Subsequently, a stratified subgroup analysis was performed to explore the prognostic value of this risk model based on the prognostic characteristics of cuproptosis-related lncRNAs. The CM patients were divided into groups according to their gender (male and female), age (<65 and ≥65), T stage (T 0-1 and T 2-4), N stage (N 0-1 and N 2-3), and stage (stage 1-2 and stage 3-4). Based on the cuproptosis-related lncRNAs prognostic signature among different clinicopathological characteristics, the Kaplan-Meier survival curve showed demonstrated that patients in the high-risk group had significant lower OS than those in the low-risk group (Figures 4A–J). Altogether, these findings demonstrate that the prognostic signature based on cuproptosis-related lncRNAs is an independent prognosis indicator that accurately predicts the prognosis of CM patients by clinicopathological parameters.

### Constructing risk model in training cohort and validation cohort

**FIGURE 5:**
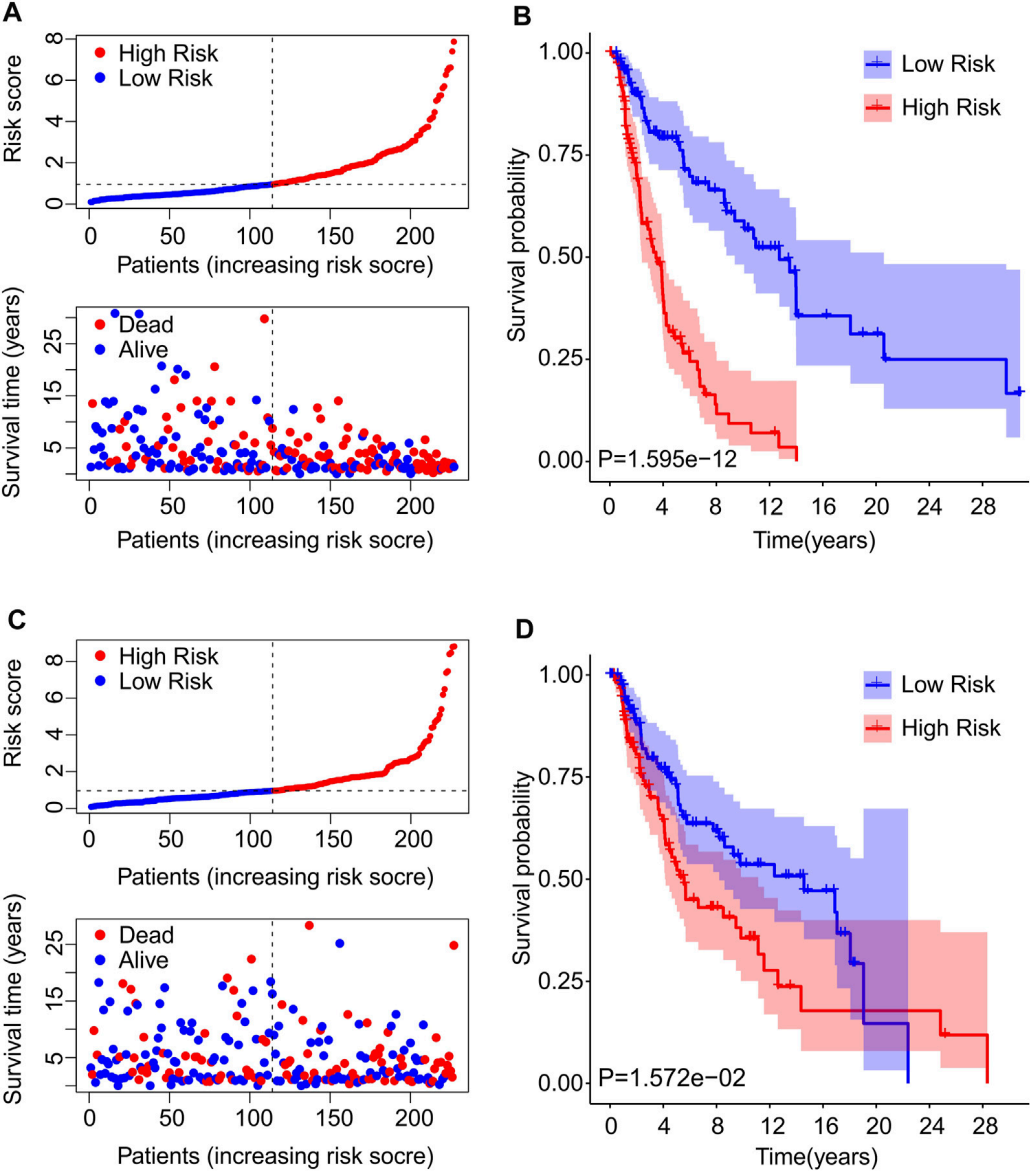
Risk model construction of training cohort and validation cohort. **(A)** The distribution of risk scores and scatter dot plot of CM patients based on the cuproptosis-related lncRNAs prognostic signature in the training cohort. **(B)** The Kaplan-Meier survival curve displayed that the overall survival rate of patients with high-risk scores was significantly shorter that those with low-risk scores in the training cohort. **(C)** The distribution of risk scores and scatter dot plot of CM patients based on the cuproptosis-related lncRNAs prognostic signature in the validation cohort. **(D)** The Kaplan-Meier survival curve showed that the overall survival rate of patients with high-risk scores is significantly shorter that those with low-risk scores in the validation cohort.

To further confirm the reliability and precision of the prognosis value of cuproptosis-based risk score, the CM patients were divided into training cohort and validation cohort based on the prognostic cuproptosis-related lncRNAs. The patients in both cohorts were divided into low and high-risk groups according to median risk scores respectively. As shown in Figure 5A, after risk score ordering, the scatter dot plot indicated that patient’s survival time in training cohort was inversely associated with risk scores. The Kaplan-Meier survival curve analysis showed that patients in the training cohort with low-risk scores had significantly higher OS than those with high-risk scores (*p* = 1.595e-12, Figure 5B). In addition, patients in the validation cohort sorted based on the risk scores and showed a similar tendency (Figure 5C). The Kaplan-Meier survival curve analysis illustrated that the patients had higher OS in the low-risk group than those in high-risk group (Figure 5D). These results demonstrate that constructing a risk model based on the prognostic cuproptosis-related lncRNAs is both accurate and feasible.

### Nomogram construction of cuproptosis-related long noncoding RNAs prognostic signature and clinicopathological characteristics

**FIGURE 6:**
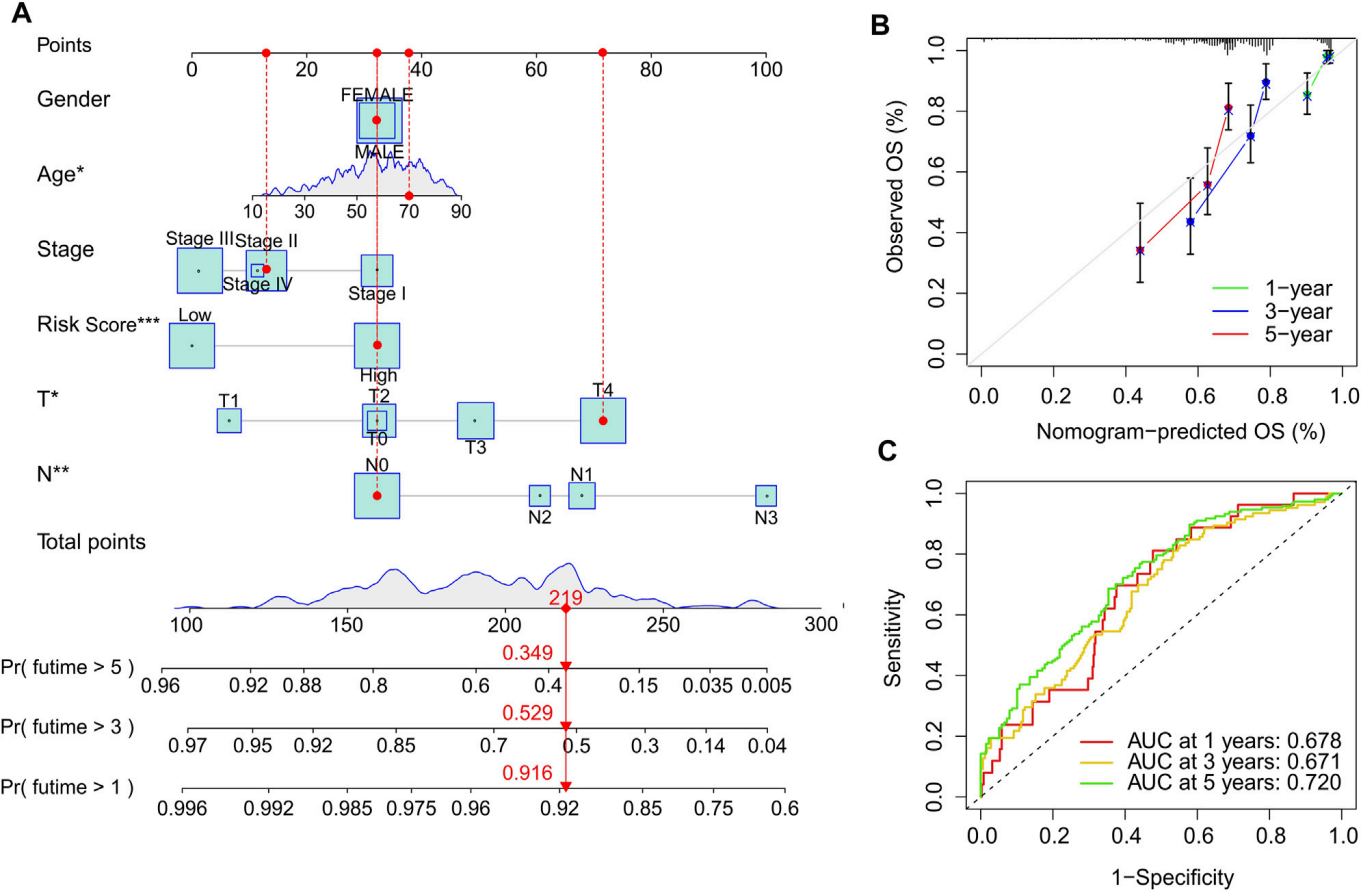
Construction nomogram of cuproptosis-related lncRNAs prognostic signatures and clinicopathological characteristics. **(A)** Nomogram construction of risk scores and other clinicopathological characteristics to predict three- and 5-year overall survival rate of CM patients. **(B)** Calibration curve revealed the accuracy between predictive power and actual survival of 1-, three- and 5-year. **(C)** Time-dependent ROC curve shows the AUC at 1-, 3-, and 5-year.

Based on the cuproptosis-related lncRNAs prognostic signature and clinicopathological characteristics, a nomogram was established to accurately predict the 1-, 3-, and 5-year survival probability of CM patients (Figure 6A). The consistency index (C-index) of the nomogram was 0.745. Additionally, the calibration curves revealed that the 1-, 3-, and 5-year survival rates predicted by nomogram showed a satisfactory consistency to the actual survival rate of CM patients (Figure 6B). The time-dependent ROC curves showed that the AUC of 1-, 3-, and 5-year was 0.678, 0.671, and 0.720, respectively, suggesting a pleasing stability of the risk model (Figure 6C). Further, a nomogram model was constructed in the validation cohort to confirm the accuracy of nomogram based on the prognostic signature and clinicopathological characteristics (Supplementary Figure S3A). The consistency index (C-index) of the nomogram was 0.754. The calibration curves indicated that the nomogram-predicted 1-, 3-, and 5-year survival rates were close to the actual survival time of CM patients (Supplementary Figure S3B). The time-dependent ROC curves showed that the AUC of 1-, 3-, and 5-year were 0.690, 0.719, and 0.799, respectively, suggesting a satisfactory stability of the risk model (Supplementary Figure 3C). These results further demonstrate that constructing a nomogram based on the cuproptosis-related lncRNAs prognostic signature to evaluate the survival probability of CM patients is accurate and reliable.

### Correlation between risk score and immune microenvironment landscape

**FIGURE 7:**
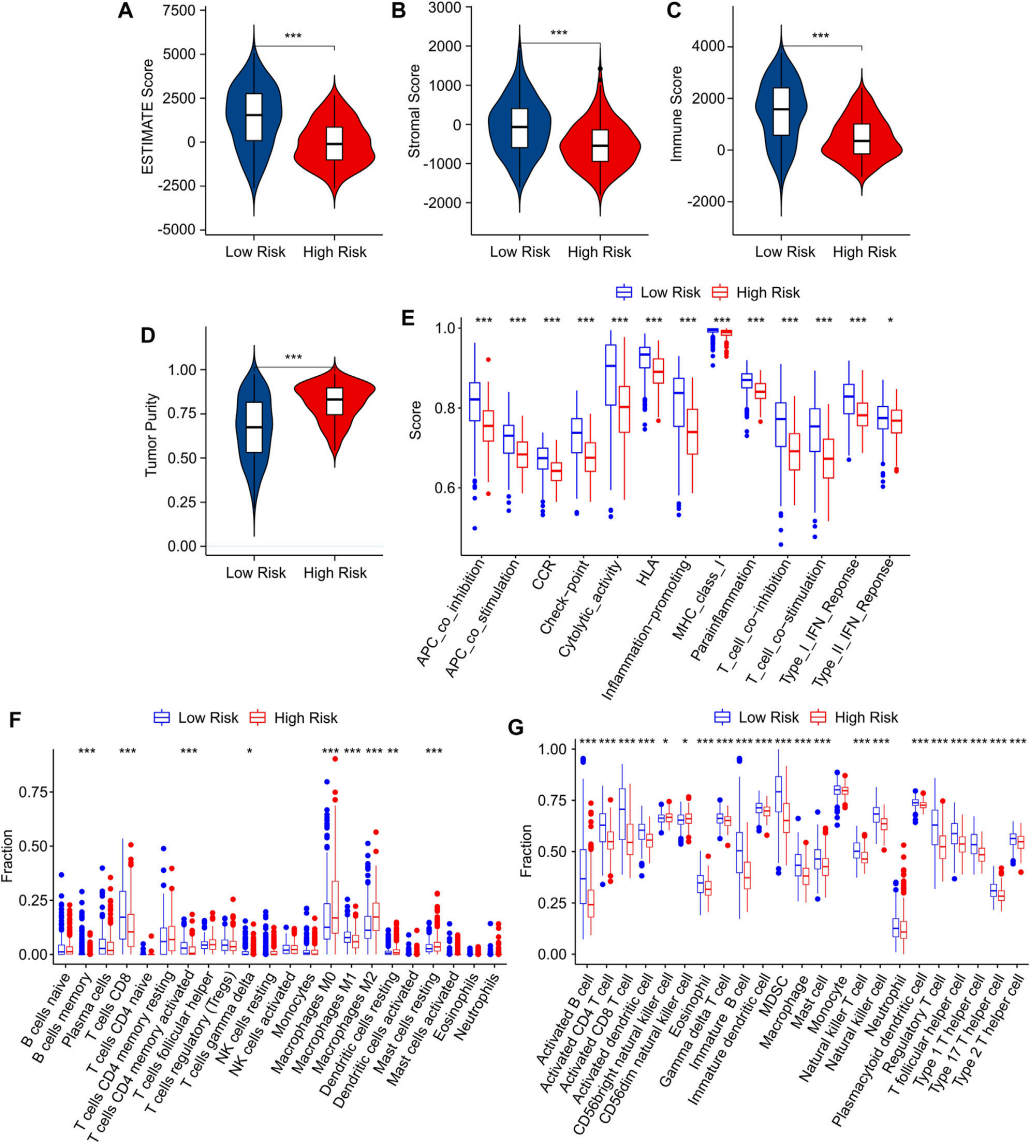
Immune infiltration characteristics of CM patients in the low- and high-risk group. **(A)** ESTIMATE score. **(B)** Stromal score. **(C)** Immune score. **(D)** Tumor purity. **(E)** The immune function score of patients in low- and high-risk groups. **(F)** The fraction of 22 immune cells in the low- and high-risk groups calculated by CIBERSORT algorithm. **(G)** The fraction of 23 immune cells in the low- and high-risk groups calculated by ssGSEA algorithm.

Tumor-infiltrating lymphocyte grade has been shown to be an independent predictor of survival and sentinel lymph node status in a variety of cancers, including CM. To investigate the TME landscape of patients in low- and high-risk groups, multiple immune assessment algorithms were performed. According to the ESTIMATE results, the patients in the low-risk group had higher ESTIMATE, immune, and stromal scores, while the tumor purity was lower (Figures 7A–D). Moreover, an immune function analysis was further conducted, and the result revealed that patients in the low-risk group had higher immune scores in most categories, such as APC, CCR, HLA, and cytolytic activity, in comparison to that of the high-risk group (Figure 7E). The CIBERSORT algorithm was used to investigate the immune infiltration of patients in low- and high-risk groups (Figure 7F). Patients in the low-risk group exhibited a markedly increased in proportion of CD8 + T cells, CD4 + memory T cells and M1 macrophages. The high-risk group promoted the proportion of M2 macrophages, dendritic cells, and mast cells, indicating a significant correlation between risk grade and immune infiltration level. Meanwhile, the results of the ssGSEA algorithm suggested that the proportion of most immune cells in the low-risk groups was much greater than in the high-risk groups, indicating that the low-risk group had a stronger immune status than the high-risk group (Figure 7G). Taken together, these results demonstrate that the risk model for cuproptosis-related lncRNAs is associated with the immune microenvironment and could indicate the immune status of CM patients.

### Risk score was associated with immunotherapy response

**FIGURE 8:**
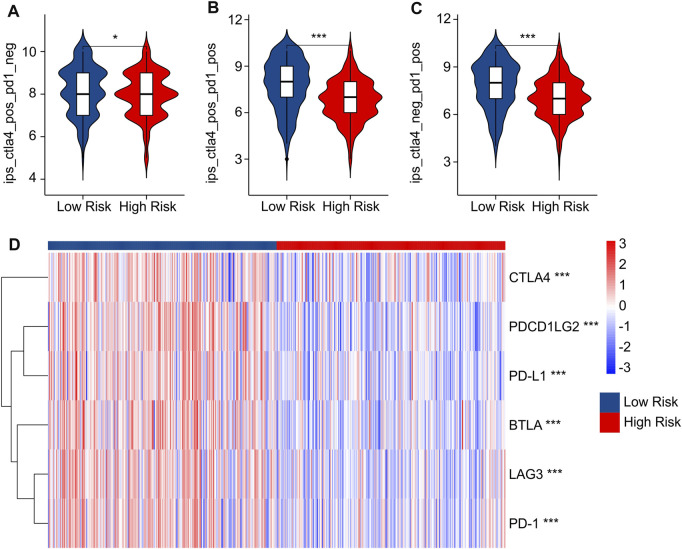
IPS scores and immune checkpoint expression of low- and high-risk groups. **(A–C)** IPS score showed the response of ICI in low- and high-risk groups. **(D)** Immune checkpoints expression in low- and high-risk groups.

Immunophenoscore (IPS) has been reported as an accurate predictor of response to anti-*CTLA-4* and anti-*PD-1*, and could indicate the response to immune checkpoint inhibitor (ICI) therapy in multitype tumors. Considering the differences in immune microenvironment status of patients in the low- and high-risk groups, the responses of immunotherapy in anti-*CTLA-4* and anti-*PD-1* were further investigated in low- and high-risk groups. IPS results indicated that the patients in low-risk group showed a promising response capacity to anti-*CTLA-4*, anti-*PD-1* and anti-*CTLA-4*/anti-*PD-1*, implying a better benefit potential for immunotherapy clinical application (Figures 8A–C). The expression of ICI in low and high-risk groups was further explored. As shown in Figure 8D, the expressions of *CTLA-4*, *PDCD1LG2*, *PD-L1*, *BTLA*, *LAG3*, and *PD-1* were significantly greater in the low-risk group. These findings demonstrated improved ICI sensitivity in low-risk patients, paving the way for the future individualized precision therapy for CM patients.

### Drug sensitivity analysis

**FIGURE 9:**
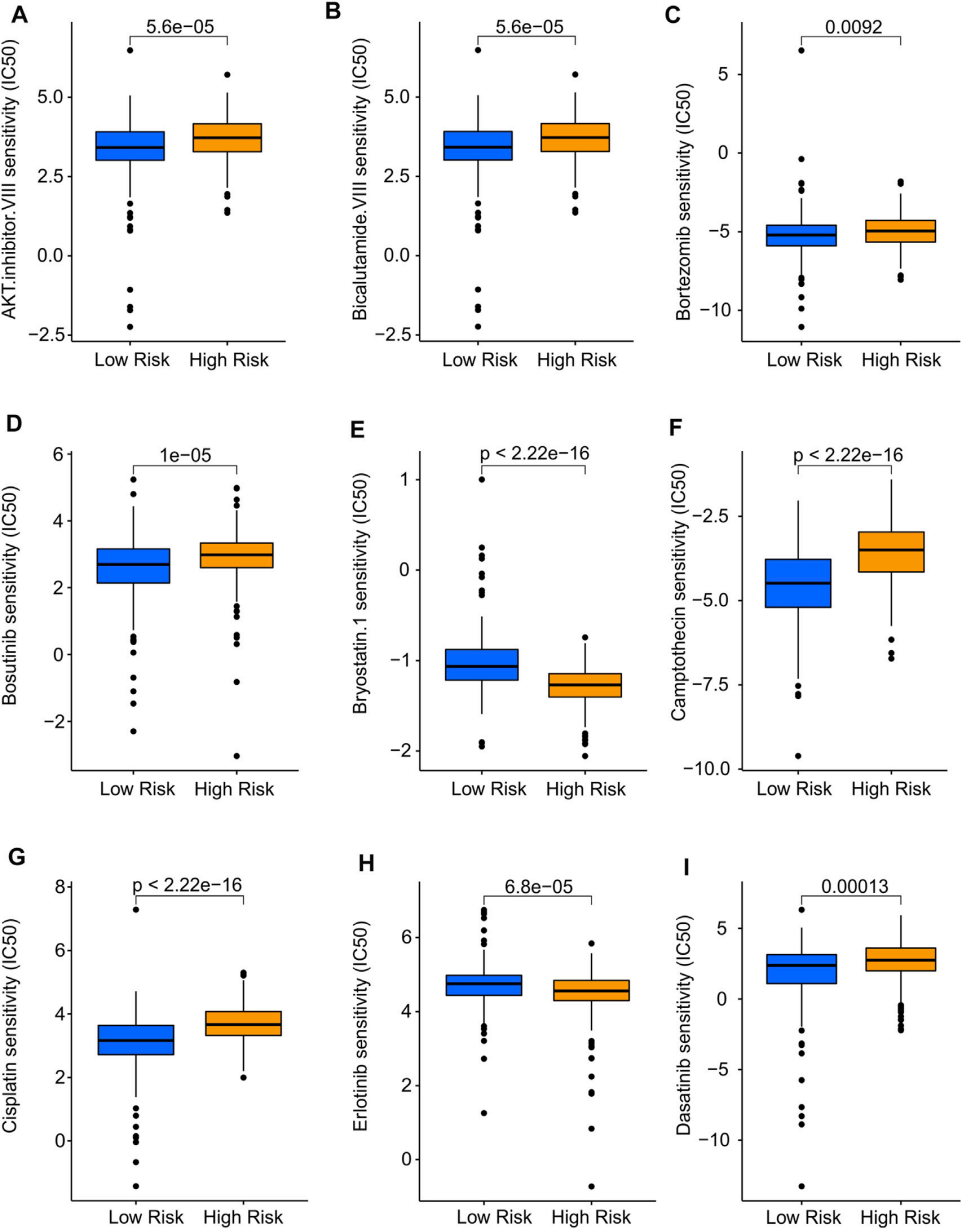
Drug sensitivity analysis of low- and high-risk groups. The distribution of IC50 showed a significant difference of patients in the low- and high-risk groups among **(A)** AKT. inhibitor. VIII, **(B)** Bicalutamide. VIII, **(C)** Bortezomib, **(D)** Bosutinib, **(E)** Bryostatin.1, **(F)** Camptothecin, **(G)** Cisplatin, **(H)** Erlotinib, and **(I)** Dasatinib. Immune-Related Signaling Pathways May Mediated the Role of Cuproptosis-Related LncRNAs in CM.

In addition to immunotherapy, targeted drug therapy is also a vital strategy for tumor treatment. Thereafter, several antineoplastic drugs were selected, which played an important role in the TME and the successful immunotherapy of CM, and the association between antineoplastic drug sensitivities and risk subgroups was further investigated. As shown in Figure 9, IC50 values of AKT, inhibitor.VIII, Dasatinib, Bortezomib, Bosutinib, Camptothecin, Cisplatin, and Bicalutamide.VIII were higher in high-risk group. Additionally, the IC50 of Bryostatin.1, Erlotinib was higher in the low-risk group than in high-risk group. Overall, these results shed light on the importance of TME in both high- and low-risk groups and provide evidence for individualized treatment of CM patients.

**FIGURE 10:**
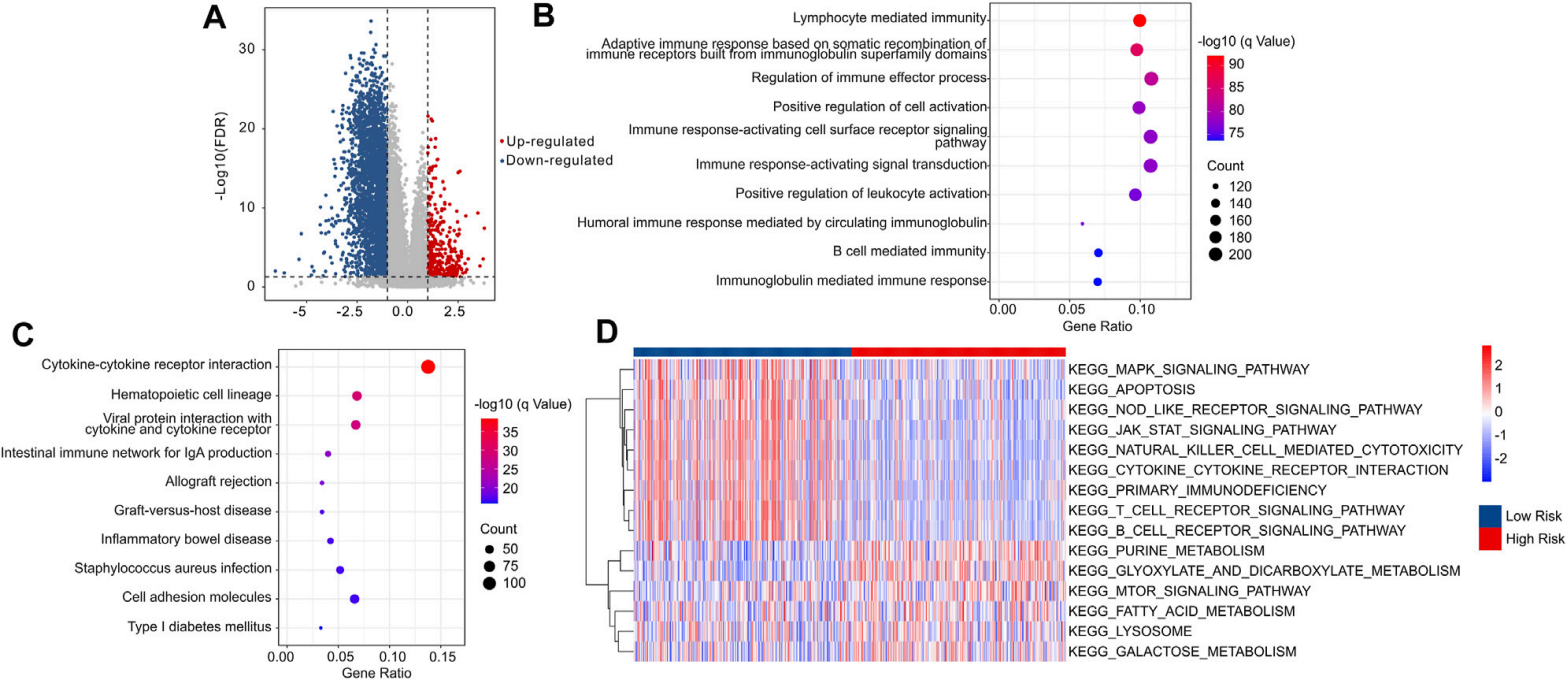
The functional enrichment analysis of DEGs between the low- and high-risk groups. **(A)** The volcano plot illustrated the DEGs in low- and high-risk groups with the threshold setting at |FC| ≥ 2 and FDR <0.05. **(B)** GO enrichment analysis suggested that the DEGs were enriched in immune-related biological processes. **(C)** KEGG enrichment analysis indicated that the enrichment signaling pathways of DEGs. **(D)** GSVA revealed the KEGG terms of each CM patients in low- and high-risk groups.

The potential molecular biology processes of differential expression genes (DEGs) in the low- and high-risk groups were investigated utilizing enrichment analysis and GSVA. Volcanogram indicated the expression of genes difference in the high-risk groups (Figure 10A). Enrichment analysis of biology process (BP) showed that the DEGs were enriched in immune-related processes such as lymphocyte mediated immunity, adaptive immune response based on somatic recombination of immune receptors built from immunoglobulin superfamily domains, and regulation of immune effector process (Figure 10B), which are in line with previous findings that risk score associated with immunotherapy response. The DEGs were shown to be abundant in cytokine-cytokine receptor interaction (Figure 10C), which is one of the major activation pathways in the immune response. Moreover, GSVA was utilized to calculate the KEGG terms. The result also showed that immune-related signaling pathways were obviously enriched in low-risk patients (Figure 10D). These findings suggest that immune-related processes may play a role in the function of cuproptosis-related lncRNAs in CM patients.

## Discussion

CM is one of the most aggressive skin malignancies, with early metastasis being the leading cause of death worldwide (Nikolaou and Stratigos, 2014). With the development of treatment strategies such as immunotherapy during the last few decades, the prognosis of CM patients has dramatically improved. However, the 5-year survival rate for patients with metastases or advanced disease remains inadequate. As a result, early detection of CM and risk stratification is critical for improving survival. Tsvetkov et al. (Tsvetkov et al., 2022) recently revealed a novel cell death mechanism (cuproptosis), that was entirely distinct from existing death mechanisms, suggesting a great potential for application in the treatment of tumors. Studies have gradually revealed that lncRNAs have crucial roles in TNM staging, tumor invasion and metastasis, leading to a great impact on the prognosis of CM (Melixetian et al., 2022). However, the role of cuproptosis-related lncRNAs in the procession of CM remains elusive. Thus, investigating cuproptosis-related lncRNAs in CM will help to uncover the mechanism of CM development. Given the probable roles of cuproptosis-related lncRNAs in the progression of cell death (Wang et al., 2022), a novel risk model was constructed to investigate the prognostic value of CM. In the current work, seven cuproptosis-related lncRNAs were discovered as being connected with overall survival rate, and a risk model was developed to accurately predict the prognosis of CM. The results indicated that the risk model constructed from seven cuproptosis-related lncRNAs may reliably predict the prognosis of CM patients by categorizing them into low- and high-risk groups. Nomogram results demonstrated that the risk model was an independent prognosis indicator that distinguished it from the other clinical characteristics. Immune infiltration landscape results revealed that the patients in the low-risk group had higher immune status than those in high-risk groups. Moreover, the patients in low-risk groups showed a promising response capacity to anti-*CTLA-4*, anti-*PD-1* and anti*-CTLA-4*/anti-*PD-1*. Functional enrichment analysis further suggested that the role of cuproptosis-related lncRNAs in the development of CM was mediated by an immunological signaling pathway. Overall, the findings in this study could provide an innovative perspective for the treatment of CM patients, stimulating the development of individualized immunotherapy and targeted therapy, and contributing to the improvement of the prognosis of patients with CM.

Cuproptosis is a newly discovered form of death, which is closely related to mitochondrial metabolic activity and has not been thoroughly studied in tumors (Tsvetkov et al., 2022). In the present study, a stratified melanoma prognosis model was established based on lncRNAs associated with cuproptosis. Tsvetko et al. have proposed that cuproptosis was associated with high mitochondrial reactivity oxidative phosphorylation (OXPHOS) (Tsvetkov et al., 2022). Despite an increasing reliance on glycolysis, it is becoming evident that many cancer cells still have functional OXPHOS (Greene et al., 2022). A report by Aminzadeh-Gohari et al. demonstrated that OXPHOS was markedly increased in melanoma cells (Aminzadeh-Gohari et al., 2020). In addition, a growing number of preclinical and clinical evidence suggests that targeting mitochondrial metabolism has anticancer benefits (Wolf, 2014; Aminzadeh-Gohari et al., 2020). OXPHOS inhibition can reduce oxygen consumption rate (OCR), reduce tumor hypoxia, and even be effective in some cancers with mtDNA mutations. Additionally, proteins involved in OXPHOS were considerably up-regulated in slow-cycling phenotypic cells (Bristot et al., 2020). Cells with a slow-cycling phenotype have intrinsic resistance, which provides a universal protective mechanism for survival from the first encounter with various cellular agents and allows additional survival time for cells to generate more durable secondary resistance mechanisms (Bristot et al., 2020). As mentioned above, overcoming inherent resistance to anticancer drugs through overcoming the slow-cycling phenotype is one of the therapeutic responses of patients with long-term malignant melanoma. Given the correlation between cuproptosis sensitivity and mitochondrial metabolic activity, these OXPHOS-upregulated drug resistant slow-cycling phenotypic cells are promising drug targets in CM.

Recent genome-wide analyses have demonstrated that lncRNAs, as regulatory factors of genome structure, have a profound impact on a variety of cell functions, such as transcription, RNA stability, and translation (Xu et al., 2021). Accumulating evidence revealed that many lncRNAs appear to have crucial roles in various cancer types, including promoting tumorigenesis (Meacham and Morrison, 2013), epithelial-mesenchymal transition (EMT) (Lamouille et al., 2014), progression (Lu et al., 2017), metastasis (Nair et al., 2020), and chemotherapy resistance (Ashrafizaveh et al., 2021). As previously indicated, lncRNAs are potential biomarkers of cancer diagnosis, prognosis and therapeutic efficacy (Yang et al., 2020), with the potential to improve anti-tumor efficacy. Several large-scale transcriptome and genomic studies have identified mal-regulated lncRNAs in melanoma (Flockhart et al., 2012). In a previous study using a sequencing database from different tissues and cancers, 339 lncRNAs were associated with CM (Iyer et al., 2015). In the current study, we found that LINC01150, EBLN3P, MIR100HG, WAC−AS1, LINC00339, and USP30−AS1 expression was elevated in the low-risk group, while AC009495.1 expression was decreased. Interestingly, elevated lncRNAs in the low-risk group are generally considered oncogenic agents in other tumor types. MIR100HG promotes EMT, tumor invasion and metastasis of colorectal cancer cells (Liu et al., 2022). LINC00339, a highly expressed lncRNA in a variety of malignant diseases, such as digestive system tumors, respiratory system tumors and nervous system tumors, is associated with the proliferation, motility and invasion of tumor cells (Wu Z. et al., 2022). WAC-AS1 could promote hepatocellular carcinoma through a glycolysis-related pathway (Xia et al., 2021). The function of lncRNA is reliant on cell environment and tissue specificity, which explains this seemingly paradoxical phenomenon. Substantial evidence suggested that the same lncRNA can function as tumor suppressor or oncogene, depending on the cell and tissue environment (Gutschner et al., 2013; Kim et al., 2018). The association between the high strong expression of these aberrant variables and low-risk stratification in CM has been re-established.

Our immune microenvironment analysis demonstrated that the cuproptosis stratified high-risk group had reduced T cell levels and considerably higher M2 macrophage expression levels, indicating a tumour suppressive immune milieu. The metabolic pathway of M1 macrophages is distinct from that of M2 macrophages. Studies have shown that the M1 macrophages display an enhanced glycolysis pathway and decreased oxygen utilization (McGettrick and O'Neill, 2020). M1 macrophages maintained their function mainly through glycolytic metabolism, despite multiple disruptions observed in the TCA cycle, fatty acid oxidation and OXPHOS also decreased in M1 macrophages. In contrast, M2 macrophages, as tumor-associated macrophages, are exposed to low oxygen, glucose, and ATP concentrations, which contribute to AMPK activation and promote an anti-inflammatory phenotype (McGettrick and O'Neill, 2020). Intense studies over the past few years have suggested that such OXPHOS-dependent M2 macrophages exhibit a complete TCA cycle, providing a substrate for the electron transport chain (ETC) (Viola et al., 2019). Importantly, the original study by Geiss et al. reported that OXPHOS could regulate the tumor immune microenvironment and that inhibition of mitochondrial OXPHOS prevents the transformation of M1 macrophages into M2 macrophages (Geiss et al., 2022). In addition, T cell activation, proliferation and function have been reported to be significantly modulated by mitochondrial metabolism (Steinert et al., 2021). Regulation of T cells via mitochondrial activation ETC and then glycolysis promotion through mTOR/AKT signaling could activate depleted T cells, enhance anti-tumor immune response and impair tumor growth. Consequently, the mitochondrial metabolism associated with cuproptosis is inextricably linked to the state of the immune microenvironment. Clinically, low LDH reflects the high dependence of cells on mitochondrial metabolism. Hence, measuring OXPHOS activity level with LDH and using copper ion carrier to mediate individual cuproptosis in patients with low LDH has potential clinical application value.

Over the past few decades, immunotherapy represented a major advance in clinical oncology, with the successful treatment of multiple cancers (Thornton et al., 2022). Growing evidence has demonstrated that immunotherapy was a key component of contemporary CM treatments (Leonardi et al., 2020). Currently, employing cancer immunotherapy to regulate the immune responses involved in CM has piqued the interest of researchers, as CM is known as one of the most highly immunogenic tumors that respond to immunological manipulations, such as the inhibition of immune checkpoints in TME (Marconcini et al., 2021). Immune checkpoint inhibitors (ICIs) are medications that activate anti-tumor responses by disrupting the inhibitory signaling to T cells (Thornton et al., 2022). Emerging studies have suggested that the immunotherapy for CM is mainly based on ICIs, such as *PD-1*, *PD-L1*, *CTLA4*, and *LAG3* (Patel et al., 2022). In our present study, IPS results indicated that the patients in the low-risk group responded positively to anti-*CTLA4*, anti-*PD-1* and anti-*CTLA-4*/anti-*PD-1*. Interestingly, our findings also revealed that anti-*PD-1* and dual-ICIs therapy (anti-*CTLA-4* + anti-*PD-1*) exhibited enhanced efficacy in patients of the low-risk group compared with single-ICI anti-*CTLA4* therapy. It has demonstrated that treatment with anti-*PD-1* monotherapy or combined anti-*CTLA-4* plus anti-*PD-1* blockade modulates the immune system differently in CM patients than in anti-CTLA-4 monotherapy. *PDCD1LG2* is the second known ligand for the *PD-1* T cell co-receptor. Limited studies have explored the potential role of *PDCD1LG2* in predicting response to ICI (Gupta et al., 2019). Taube and colleagues have reported that the areas of tumor cell *PDCD1LG2* expression were adjacent to immune infiltrates in the CM case. *PDCD1LG2* expression was geographically associated with *PD-L1*, consistent with its known up-regulation by inflammatory cytokines including interferon-gamma which also drives *PD-L1* expression (Taube et al., 2014). B and T Lymphocyte Attenuator (*BTLA*), as its name described, is a co-inhibitory receptor that could induce immunosuppression by inhibiting B and T cell activation and proliferation. As a third inhibitory receptor on T lymphocytes, *BTLA* is related to *PD-1* and *CTLA-4* in terms of its structure and function (Watanabe et al., 2003; Fallon et al., 2018). *LAG-3* (Lymphocyte activation gene-3), an immune checkpoint pathway, particularly via its role in negatively regulating the activation, proliferation, and effector function of both CD8^+^ and CD4^+^ T-cells as well as mediating immune tolerance (Pennock and Chow, 2015). In the present study, the expressions of *CTLA-4*, *PDCD1LG2*, *PD-L1*, *BTLA*, *LAG3*, and *PD-1* were significantly higher in low-risk CM group. These results illustrated that the patients in different risk subtypes respond differently to immunotherapy, providing an innovative approach for the individualized treatment of CM patients.

The identification of different risk stratification groups’ signaling pathways may lead to a better understanding of the potential molecular biology process of CM. Enrichment analysis suggested that the differentially expressed genes between the low- and high-risk groups were mainly enriched in immune-related processes. Furthermore, pathway analysis revealed enrichment of DEGs in KEGG pathways of cytokine-cytokine receptor interaction. The cytokine–cytokine receptor interaction is a major contributor to cellular inflammatory response, which in turn is a critical component in the CM process (Karlsson et al., 2021). It is noteworthy that patients with low-risk scores had a better immune status as revealed by GSVA. Taken together, our findings indicated that an immune-related signaling pathway was an important aspect affecting CM progression, which was regulated by cuproptosis-related lncRNAs. From this novel perspective, the present study has elucidated the role of cuproptosis-related lncRNAs in the immune regulation of CM, thus offering a new basis for future treatment of CM.

In the present study, we developed a prognostic risk model based on lncRNAs associated with cuproptosis and effectively differentiated CM into low- and high-risk groups. Our analysis clearly shows a significant correlation between this model and prognosis. In addition, immune cell infiltration analysis and lncRNAs functional enrichment analysis were carried out, which preliminarily demonstrated the correlation between the CM model and the immunosuppressive microenvironment. This work will lay a preliminary foundation for further experimental verification.

## Data Availability

The original contributions presented in the study are included in the article/Supplementary Material, further inquiries can be directed to the corresponding authors.

## Abbreviations

CM, cutaneous melanoma; LncRNAs, long noncoding RNAs; OS, overall survival; IPS, immunophenoscore; GSVA, gene set variation analysis; ICB, immune checkpoint blockade; TCA, tricarboxylic acid; LDH, lactate dehydrogenase; TME, tumor microenvironment; TIME, tumor immune microenvironment; TCGA, The Cancer Genome Atlas; LASSO, least absolute shrinkage and selection operator; PCA, principal component analysis; ROC, operating characteristic; DEG, differential expression gene; BP, biology process; KEGG, Kyoto Encyclopedia of Genes and Genomes; HR, hazard ratio; CTLA-4, cytotoxic T lymphocyte-associated antigen-4; PD-1, programmed cell death protein 1; OXPHOS, oxidative phosphorylation; OCR, oxygen consumption rate; EMT, epithelial-mesenchymal transition; ETC, electron transport chain.
