# Supplementary material for: A novel risk model based on cuproptosis-related lncRNAs predicted prognosis and indicated immune microenvironment landscape of patients with cutaneous melanoma
Source: Front Genet. 2022 Jul 22;13:959456. doi: 10.3389/fgene.2022.959456 (PMC9354044; doi:10.3389/fgene.2022.959456)
Supplement: Supplementary file 1 [file DataSheet1.docx]

Supplementary Table 1. Univariate Cox regression of clinical factors and risk scores.

| Variates | HR | HR.95L | HR.95H | *P* Value |
| --- | --- | --- | --- | --- |
| Age | 1.0198 | 1.0089 | 1.0308 | 0.000358 |
| Gender | 1.0349 | 0.7377 | 1.4517 | 0.842569 |
| Stage | 1.4726 | 1.2172 | 1.7816 | 6.81E-05 |
| T | 1.4454 | 1.2425 | 1.6813 | 1.80E-06 |
| N | 1.4431 | 1.2335 | 1.6882 | 4.60E-06 |
| Risk Score | 2.2121 | 1.8061 | 2.7094 | 1.67E-14 |

Abbreviation HR: Hazard Ratio

Supplementary Table 2. Multivariate Cox regression of clinical factors and risk scores.

| Variates | HR | HR.95L | HR.95H | *P* Value |
| --- | --- | --- | --- | --- |
| Age | 1.00718 | 0.996111 | 1.018372 | 0.204498 |
| Gender | 1.036935 | 0.735594 | 1.461722 | 0.835979 |
| Stage | 0.893026 | 0.642414 | 1.241405 | 0.500805 |
| T | 1.341774 | 1.130955 | 1.591891 | 0.000749 |
| N | 1.584016 | 1.240492 | 2.022671 | 0.000226 |
| Risk Score | 1.872236 | 1.521355 | 2.304041 | 3.17E-09 |

Abbreviation HR: Hazard Ratio


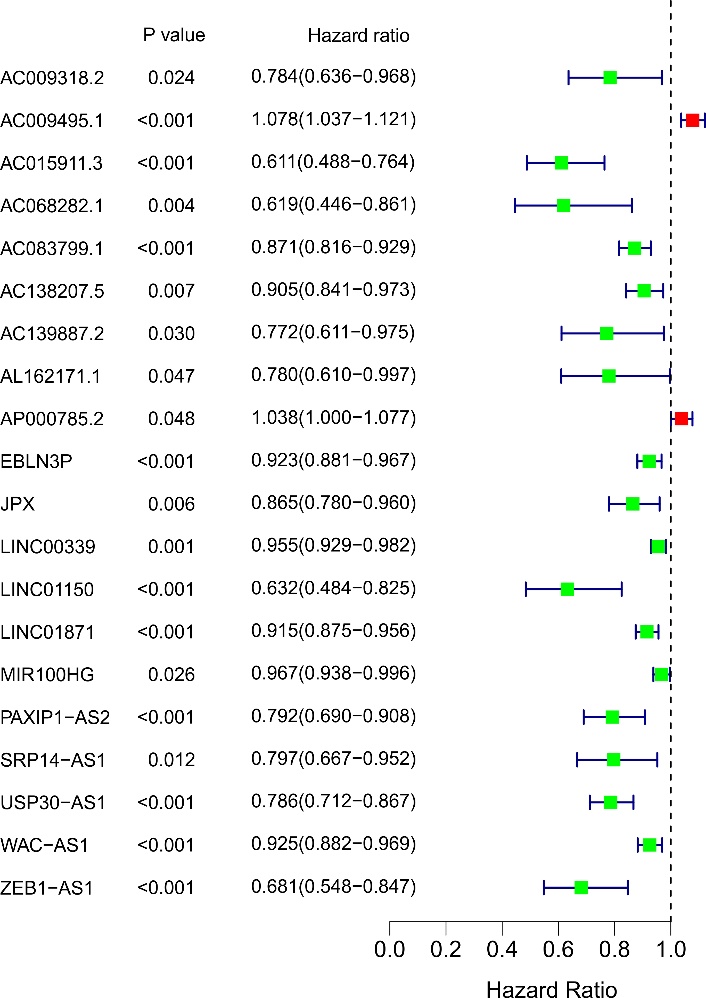


Supplementary Figure 1. Univariate Cox regression analysis of 20 prognostic cuproptosis-related lncRNAs.


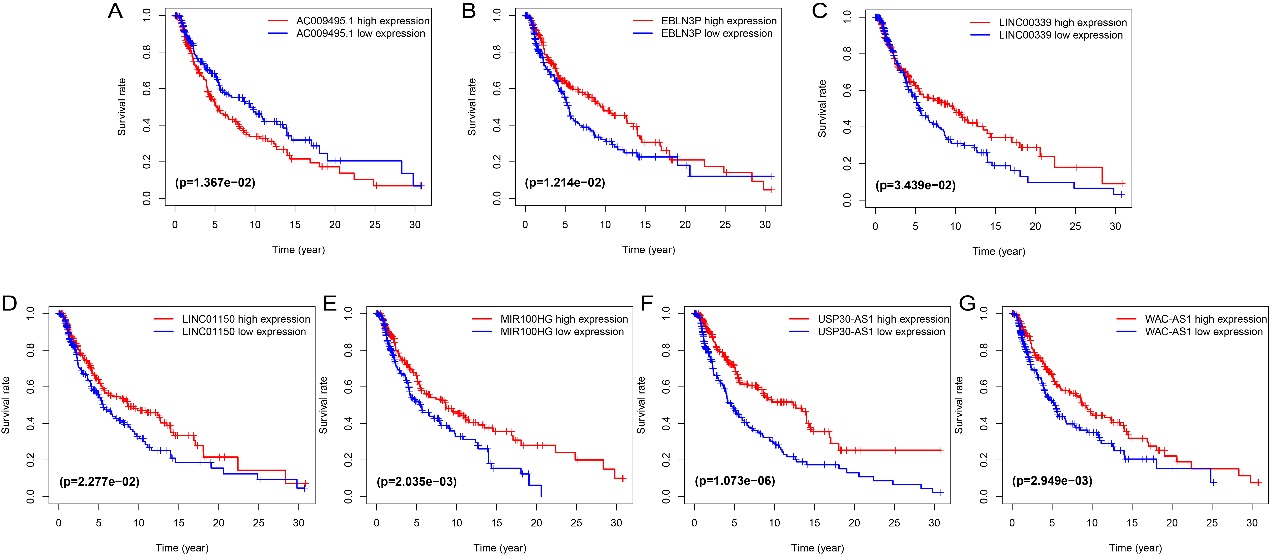


Supplementary Figure 2. Survival curve analysis of 7 cuproptosis-related lncRNAs. The survival curve of (A) AC009495.1, (B) EBLN3P, (C) LINC00339, (D) LINC01150, (E) MIR100HG, (F) USP30-AS1, and (G) WAC-AS1.


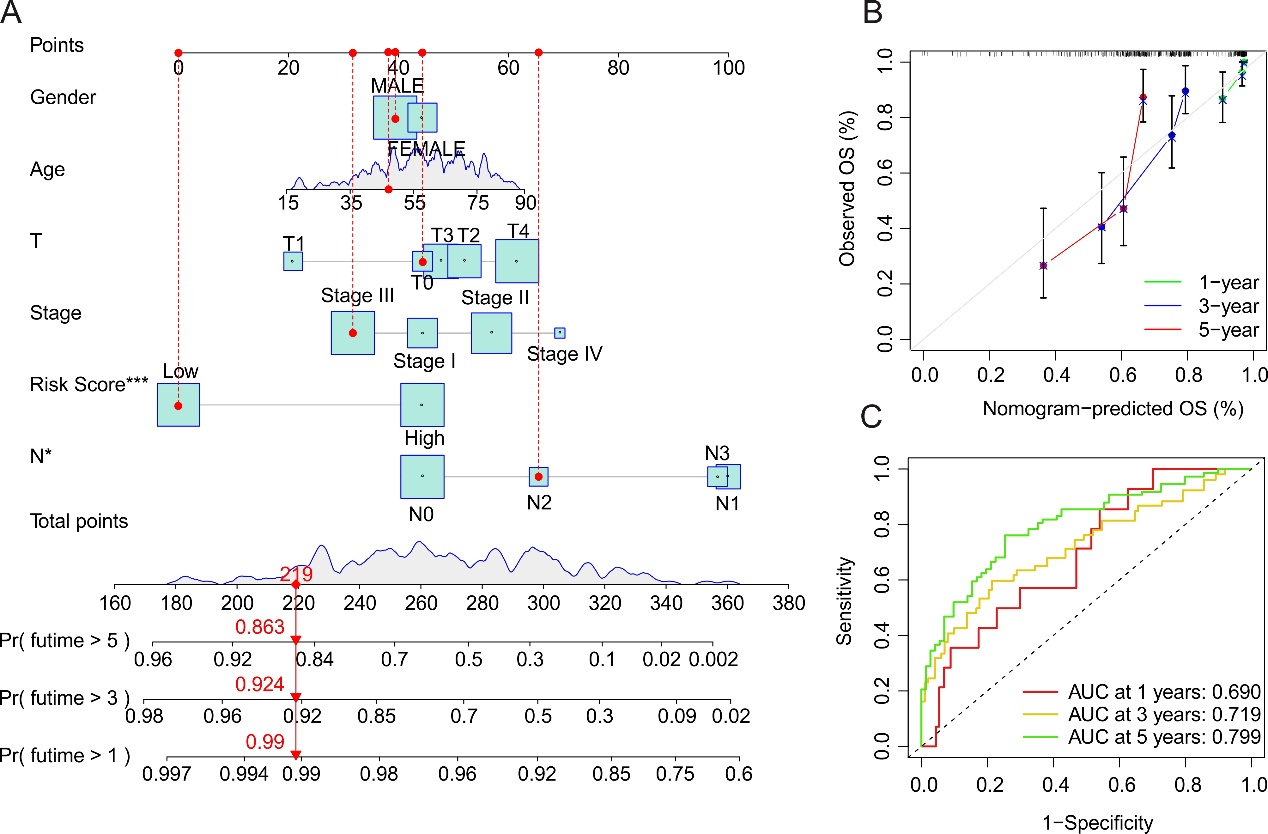


Supplementary Figure 3. Construction nomogram of cuproptosis-related lncRNAs prognostic signatures and clinicopathological characteristics in validation cohort. (A) Nomogram construction of risk scores and other clinicopathological characteristics to predict 3- and 5-year overall survival rate of CM patients. (B) Calibration curve revealed the accuracy between predictive power and actual survival of 1-, 3- and 5-year. (C) Time-dependent ROC curve shows the AUC at 1-, 3-, and 5-year.
